# *Camelina sativa* meal hydrolysate as sustainable biomass for the production of carotenoids by *Rhodosporidium toruloides*

**DOI:** 10.1186/s13068-020-01682-3

**Published:** 2020-03-12

**Authors:** Stefano Bertacchi, Maurizio Bettiga, Danilo Porro, Paola Branduardi

**Affiliations:** 1 Department of Biotechnology and Biosciences, University of Milano-Bicocca, Piazza della Scienza 2, 20126 Milan, Italy; 2 Division of Industrial Biotechnology, Department of Biology and Biological Engineering, Chalmers University of Technology, Kemivägen 10, 412 96 Gothenburg, Sweden; 3 EviKrets Biobased Processes Consultants, Lunnavägen 87, 42834 Landvetter, Sweden

**Keywords:** Bio-based products, Renewable resources, Biorefinery, *Camelina* meal, Enzymatic hydrolysis, *Rhodosporidium toruloides*, Carotenoids, Separate hydrolysis and fermentation (SHF), Simultaneous saccharification and fermentation (SSF)

## Abstract

**Background:**

As the circular economy advocates a near total waste reduction, the industry has shown an increased interest toward the exploitation of various residual biomasses. The origin and availability of biomass used as feedstock strongly affect the sustainability of biorefineries, where it is converted in energy and chemicals. Here, we explored the valorization of *Camelina* meal, the leftover residue from *Camelina sativa* oil extraction. In fact, in addition to *Camelina* meal use as animal feed, there is an increasing interest in further valorizing its macromolecular content or its nutritional value.

**Results:**

*Camelina* meal hydrolysates were used as nutrient and energy sources for the fermentation of the carotenoid-producing yeast *Rhodosporidium toruloides* in shake flasks. Total acid hydrolysis revealed that carbohydrates accounted for a maximum of 31 ± 1.0% of *Camelina* meal. However, because acid hydrolysis is not optimal for subsequent microbial fermentation, an enzymatic hydrolysis protocol was assessed, yielding a maximum sugar recovery of 53.3%. Separate hydrolysis and fermentation (SHF), simultaneous saccharification and fermentation (SSF), and SSF preceded by presaccharification of *Camelina* meal hydrolysate produced 5 ± 0.7, 16 ± 1.9, and 13 ± 2.6 mg/L of carotenoids, respectively. Importantly, the presence of water-insoluble solids, which normally inhibit microbial growth, correlated with a higher titer of carotenoids, suggesting that the latter could act as scavengers.

**Conclusions:**

This study paves the way for the exploitation of *Camelina* meal as feedstock in biorefinery processes. The process under development provides an example of how different final products can be obtained from this side stream, such as pure carotenoids and carotenoid-enriched *Camelina* meal, can potentially increase the initial value of the source material. The obtained data will help assess the feasibility of using *Camelina* meal to generate high value-added products.

## Background

The continued use of fossil resources poses an ecological, economic, and political problem that has sparked the search for alternative sources of energy, chemicals, and materials. Biorefineries, which transform biomass into energy and chemicals, offer a possible answer, particularly in the form of microbial cell factories. The sustainability of biorefineries is strongly related to the origin, availability, and market of biomass. For example, edible crops have been exploited for decades as feedstocks for the production of several fine and bulk chemicals. However, environmental and social issues, caused by direct or indirect competition with the food sector, discourage the use of agricultural products and land for large-scale production of commodities [1]. At the same time, the existing linear economy’s logic of “take, make, dispose” is generating a large amount of waste, including organic matter. For these reasons, biorefineries based on residual biomasses have drawn increasing scientific and industrial interest. Microbial cell factories are especially attractive; however, conventional pretreatments and saccharification processes of residual biomasses often release toxic compounds that can impair microbial growth and synthesis of the target product [2]. These issues need to be factored in when developing robust biorefineries capable of generating high-value molecules from low-cost substrates.

The growing use of oilseed crops for food and biofuels is leading to a surplus of process leftovers that are currently used mainly as animal feed [3] owing to their protein, carbohydrate, and fiber content. A good example is *Camelina* meal (or cake), the main by-product of oil extraction from *Camelina sativa* seeds [4–8], which is a common supplement of cattle and poultry diet. However, the rich composition and relatively low cost ($0.25/kg) of *Camelina* meal [9], make it attractive for the development of sustainable bio-based processes that would either further valorize its macromolecular components or increase its nutritional value in animal feed. So far, only Mohammad et al. [10] have attempted to use *Camelina* meal, mixed with other *Camelina*-derived sugars, for the production of bioethanol, a low value-added molecule, by *Saccharomyces cerevisiae*. To improve the economic viability of the process, the present study assessed the microbial biotransformation of *Camelina* meal into carotenoids as high value-added products.

The global market value for carotenoids was estimated to be $1.5B in 2017 and is expected to reach $2.0B by 2022, with a compound annual growth rate of 5.7% [11–13]. Carotenoids are found mainly in animal feed (41% of total revenue), followed by food and dietary supplements owing to their beneficial effect on human health [12, 14]. Ruminants are entirely dependent on feed as a source of carotenoids, since they cannot produce them on their own [3]. Chemical synthesis of carotenoids from synthetic resources meets 80–90% of the market needs, but the increasing demand for naturally produced molecules has sparked the search for new, preferably vegetal sources [12]. β-Carotene alone has a market value of $246.2M. Natural β-carotene can be extracted from carrots and fruits of oil palm, but recent attempts have demonstrated the commercial production of β-carotene in microbial cell factories employing the microalga *Dunaliella salina* or the filamentous fungus *Blakeslea trispora* [12]. Unfortunately, algal carotenoid production is generally expensive and requires large areas for cultivation [15, 16], whereas filamentous fungi are frequently characterized by slow growth and a multicellular nature that may impair productivity [17]. Yeasts could potentially improve the overall sustainability of the process. In particular, the oleaginous yeast *Rhodosporidium* (*Rhodotorula*) *toruloides*, also known as “pink yeast”, naturally accumulates carotenes and xanthophylls, such as β-carotene, torulene, and torularhodin [16, 18, 19]. *R. toruloides* can use different sugars, such as glucose, cellobiose, sucrose, mannose, xylose, arabinose, and galacturonic acid, as main carbon sources [20]. In addition, *R. toruloides* converts complex substrates, such as carob pulp syrup, sugarcane bagasse, corn stover, and food wastes, into lipids and/or carotenoids [21–24]. Therefore, this yeast is a good candidate for the development of second-generation biorefineries.

To produce carotenoids in *R. toruloides*, *Camelina* meal was first saccharified by enzymatic hydrolysis. Then, the released sugars were used as feedstock in separate hydrolysis and fermentation (SHF). An alternative simultaneous saccharification and fermentation (SSF) process was also assessed. Results indicate that *Camelina* meal and *R. toruloides* can be used for the development of a novel bio-based process for carotenoids production. Moreover, the obtained data will facilitate further optimization of process conditions.

## Results and discussion

### Evaluation of total sugar content in *Camelina* meal and optimization of enzymatic hydrolysis

The content of water, insoluble components, acetic acid, and sugars in *Camelina* meal was quantified following acid hydrolysis. Acetic acid and sugars were analyzed also after enzymatic hydrolysis, as no enough data exist in literature to assess the use of *Camelina* meal as substrate for microorganisms. As shown in Table 1, almost one-third (31%) of *Camelina* meal was composed of sugars. Of these, glucose and fructose accounted for more than two-thirds (w/w) as revealed by high-performance liquid chromatography (HPLC) analysis. Even though acid hydrolysis can be suitable for saccharification, its use is limited by the low final pH, which needs to be neutralized before sugars are added to the cells, and by the release of inhibitory compounds such as furfurals [2, 25]. These limitations negatively affect the sustainability of the overall biorefinery process in terms of use and disposal of acid solutions [25]. Therefore, to release monomeric sugars from their polymeric form, an enzymatic instead of an acid hydrolysis was performed under different test conditions (see below). The other main components of *Camelina* meal, as reviewed by [7], are crude proteins and crude fats, which account for 35.2–46.9% and 4.9–11.9% of total biomass, respectively, as well as micronutrients such as vitamins; whereas the insoluble fraction is composed mainly of lignin and ashes. Based on these data, we hypothesized that *Camelina* meal could be a suitable substrate for the growth of *R. toruloides* and carotenoid production.

**Table 1:** *Camelina* meal hydrolysate composition following acid treatment

*Camelina* meal composition	
Measured component	Percentage (w/w)
Water	9 ± 1.8%
Acetate	11 ± 1.4%
Insoluble fraction	13 ± 1.4%
Sugars of which	31 ± 1.0%
Glucose	16 ± 0.9%
Fructose	8.3 ± 0.0%
Arabinose	6.9 ± 0.0%
Crude protein	35.2–46.9% [7]
Crude fat	4.9–11.9% [7]

Values are the means of three independent experiments

**Fig. 1:**
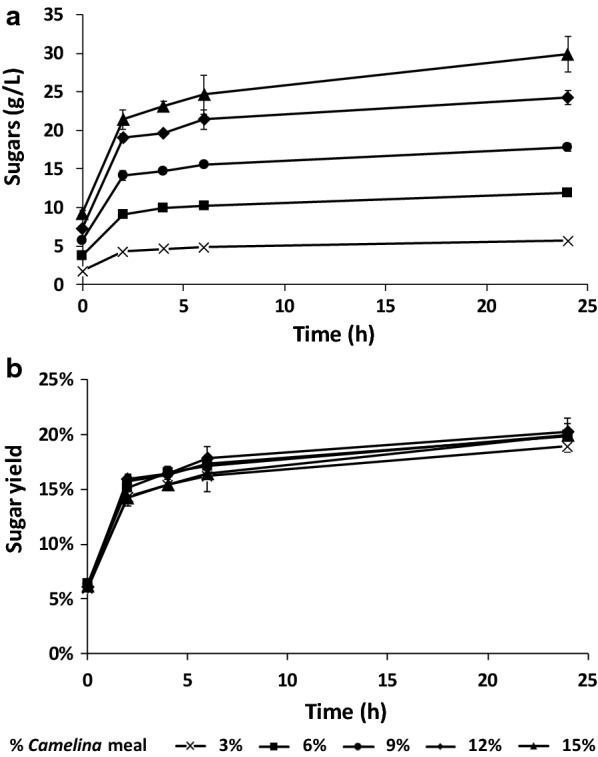
Effect of enzymatic hydrolysis with the NS22119 cocktail (11.9% w/w_*Camelina* meal_) on different *Camelina* meal concentrations. Time course of sugar released (**a**) and sugar yield from biomass (**b**). Values are the means of three independent experiments

Enzymatic hydrolysis can be performed under conditions that are generally more compatible with subsequent growth of mesophilic microbial cell factories. Moreover, it can take advantage of a broad range of commercially available enzymatic cocktails [26, 27]. Here, this step was optimized using the commercial cocktail NS22119 (Novozymes A/S), which can release both hexose and pentose sugars. Different initial concentrations of *Camelina* meal were tested to determine the effect of solids loading on sugar release. After autoclaving, the measured pH was 5.5, which was compatible with enzymatic catalysis, as NS22119 is supposed to retain up to 90% of its maximum activity at this pH, according to the indications of the producer. Remarkably, the pH remained constant until the end of hydrolysis, which reduced both the economic and environmental impact of the procedure, as neither neutralization nor additional buffer was required. As shown in Fig. 1a, pretreatment of biomass by autoclaving resulted in the initial concentration of released sugars to range from 1.8 ± 0.03 to 9 ± 0.3 g/L. The values reflected the amount of biomass loaded at the beginning of the experiment. After enzymatic treatment (11.9% w/w_*Camelina* meal_), the concentration of free sugars rose to at least double the initial amount, independently of the quantity of loaded biomass (Fig. 1a). No additional release of sugar was detectable over time from negative control samples, in which 3% or 15% of the initial biomass but no enzyme was incubated in a shaking water bath at 50 °C (Additional file 1: Figure S1). The sugar titer increased in the presence of enzymes in a linear manner (*R*^2^ = 0.98, *p* < 0,001, calculated with *R*) until 24 h in respect to the initial quantity of biomass. Hence, the yield of sugars released by enzymatic hydrolysis was constant regardless the concentration of *Camelina* meal (Fig. 1b) and the maximum yield of sugars over total biomass was 20% after 24 h. Considering the original amount of carbohydrates, a sugar recovery of 65% was calculated (see “Calculations and statistical analyses” section), which is in accordance with commonly reported values for lignocellulose enzymatic hydrolysis [28, 29].

**Fig. 2:**
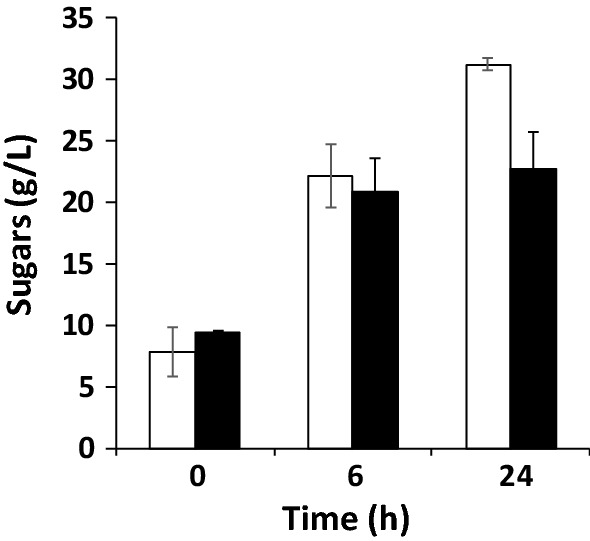
Enzymatic hydrolysis of 15% *Camelina* meal. Sugars released by supplementation with an additional pulse of NS22119 cocktail (11.9% w/w_*Camelina* meal_) after 6 h of hydrolysis (white bar) or with a starting double enzymatic cocktail dose (23.8% w/w_*Camelina* meal_) (black bar). Values are the means of three independent experiments

Based on these data, successive experiments were performed using *Camelina* meal at the maximum tested solids loading (15% w/v). To determine if sugar recovery could be further improved in spite of a possible inhibition of enzymatic activity by released products or by biomass itself, two different strategies were designed. In one, the initial quantity of enzymes was doubled from 11.9 to 23.8% w/w_*Camelina* meal_; in the other, the mixture was pulsed with a second dose of enzymes (11.9% w/w_*Camelina* meal_), thus doubling the total amount, after 6 h of hydrolysis. When the first strategy using double the amount of enzymes was applied, the quantity of sugars released from *Camelina* meal (Fig. 2, black bars) did not differ significantly from that of a single enzyme dose (Fig. 1a). A similar result was obtained when the second strategy, based on an additional pulse of enzymatic cocktail, after 6 h of hydrolysis was applied (Fig. 2, white bars). These findings indicate that incomplete saccharification is related more to the intrinsic accessibility of polysaccharides in the biomass than to limitations in catalytic activity. They also suggest that the initial procedure was the preferred one, as it minimized the use of enzymes and thus the overall cost of the process. The enzymatic cocktail exhibited greatest activity during the first hours of hydrolysis; prolonging incubation beyond 6 h to 24 h improved sugar titer by only 20%. Therefore, the conditions for enzymatic hydrolysis of *Camelina* meal were as follows: 15% w/w solids loading, 11.9% w/w_*Camelina* meal_ of enzymatic cocktail NS22119, reaction time of 6 h, operative temperature of 50 °C, and initial pH of 5.5. As underlined before, the pH of the reaction mixture remained constant over time and was conveniently closer to the optimum reported for carotenoids accumulation in *R. toruloides* (pH 5) than to the value suitable for lipid production (pH 4) [30].

The above settings allowed for about 25 g/L of monomeric sugars to be released and with a sugar recovery of 53.3%. The fraction of residual non-hydrolyzed carbohydrates could be considered as an added value to the final product, since a *Camelina* meal enriched in carotenoids by fermentation of *R. toruloides* would still contain fibers of nutritional value.

### Inhibitory compounds in *Camelina* meal hydrolysate

Compared with traditional acid treatment, enzymatic hydrolysis is efficacious in releasing sugars from lignocelluloses and minimizing the accumulation of inhibitory compounds [31, 32]. Nevertheless, there are some drawbacks related to other compounds detached from these complex matrices [2, 20]. Acetic acid is the most common inhibitor released by hydrolysis of the hemicellulose fraction composing lignocellulosic biomasses. Acetic acid can easily impair microbial growth and metabolism due to its generic and specific toxicity [33], reducing the key performance indicators of the production process [2, 33, 34]. Nevertheless, the toxicity of acetic acid is greater at low pH; extracellular pH values higher than its pK_a_ (4.76) reduce its diffusion across the membrane and, therefore, the cellular damage it could trigger [35, 36]. In the present study, the operative pH (5.5) was higher than the pK_a_ of acetic acid, thus lowering the detrimental effect of this molecule on cells. Moreover, *R. toruloides* has been shown to withstand acetic acid when added to defined media or even as the sole carbon source at up to 20 g/L at pH 6 [37–39]. During enzymatic hydrolysis, acetic acid titer increased, reaching 1.8 ± 0.01 g/L after 24 h from the start (Additional file 1: Figure S2). This amount has been described as bearable by diverse yeasts [34, 37, 38] including *R. toruloides*.

Considering the above constrains, *Camelina* meal hydrolysate appears to be a suitable feedstock for yeast cell factory-based biorefineries, which would enable the exploitation of yeast biodiversity and engineering strategies to obtain different products of interest.

### Carotenoids production from *Camelina* meal hydrolysate in SHF and SSF processes

**Fig. 3:**
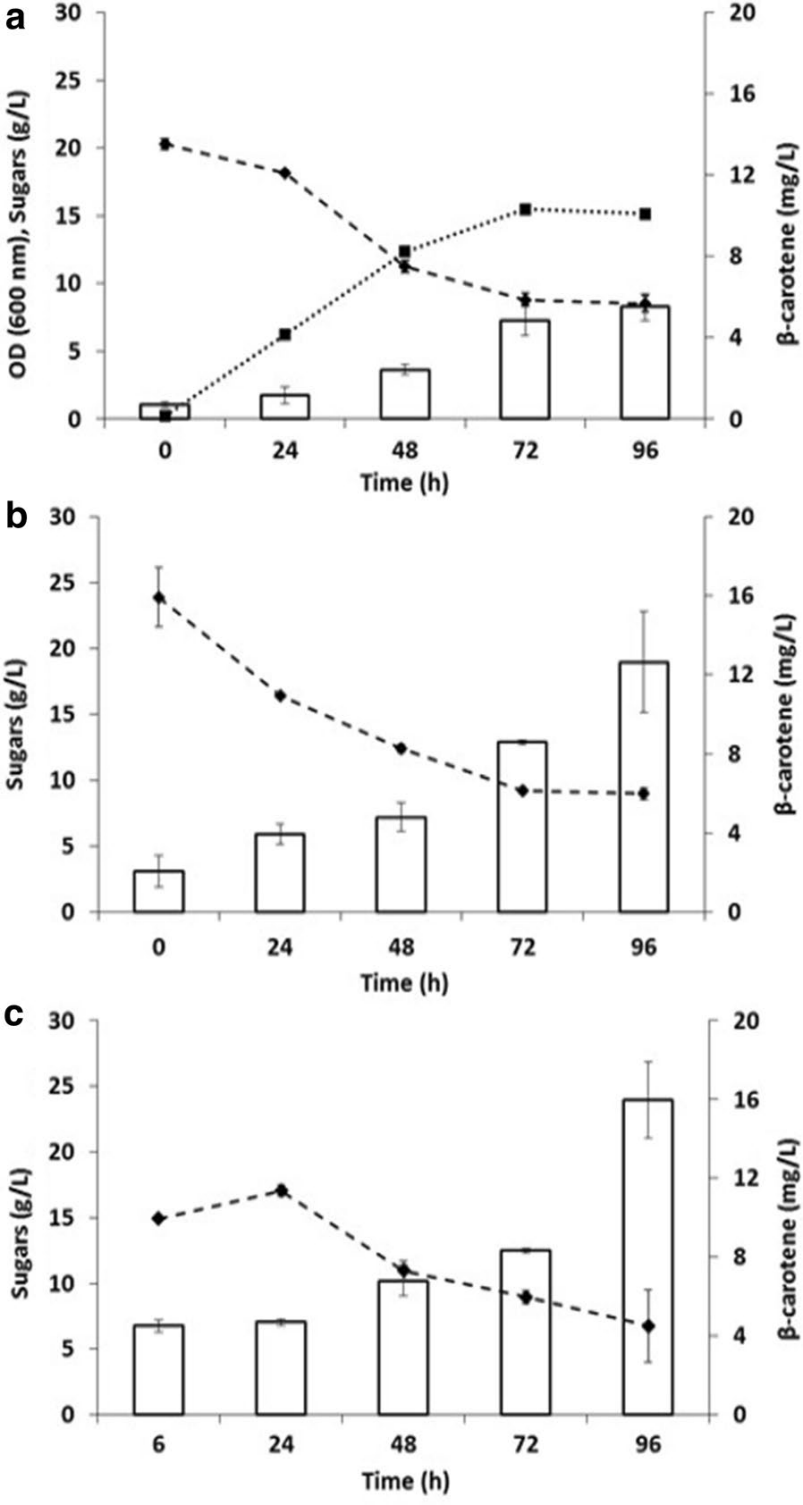
*R. toruloides* production of carotenoids from 15% *Camelina* meal hydrolysate. OD (dotted line), sugars consumption (dashed line), and β-carotene production (white bars) by *R. toruloides* subjected to different processes: SHF (**a**), SSF + presaccharification (**b**), and SSF (**c**). Values are the means of three independent experiments

Having established a protocol for obtaining *Camelina* meal hydrolysate, carotenoids production by SHF was investigated. In the SHF experiment, medium consisted of clarified supernatant collected after 6 h of enzymatic hydrolysis. This medium was sufficient to sustain *R. toruloides* growth, as indicated by the accumulation of biomass and consumption of sugar (Fig. 3a, dotted and dashed lines, respectively). The accumulation of carotenoids increased over time, reaching 5 ± 0.7 mg/L after 96 h of fermentation (Fig. 3a, white bars), with a yield on consumed sugars of 0.034% (w/w) and on maximum quantity of sugars per biomass of 0.011% (w/w). These data are in accordance with previous reports that *R. toruloides* and carotenogenic microorganisms in general produce carotenoids mostly in response to stressful or sub-optimal conditions such as stationary phase [17, 18, 40, 41]. The period of 96 h was chosen mainly to allow comparison with previous studies, whereby *R. toruloides* was provided with defined media or other/different residual biomasses (Table 2). After 96 h, fewer carotenoids could be extracted from the cells (Additional file 1: Figure S3), which could correlate with the export/release of carotenoids from the cells or with an imbalance of nutrients that might promote their consumption/corruption. The carotenoid production achieved here by SHF was competitive with *R. toruloides* grown in shake flasks and supplemented with other complex matrices (Table 2).

**Table 2:** Carotenoids production by *R. toruloides*

*R. toruloides* strain	Substrate	Time (h)	β-Carotene (mg/L)	References
ATCC 204091	WE^1^	72	62 ± 1.70	[23]
	MM^2^	100	57 ± 2.18	
ATCC 10788	YPG^a^	288	3.6	[16]
AS 2.1389			4.3	
CBS 5490			6.8	
CCT 0783	SCBH^b^	72	1.2 ± 0.1	[21]
	cSCBH	94	2.18 ± 0.2	
NCYC 921 (alias ATCC 10788)	CPS100^c^	48	0.41	[22]
	CPS75		0.47	
	SCM100^d^		0.04	
	SCM75		0.18	
DSM 4444	CM SHF^e^	96	5.5 ± 0.7	This study
	CM SSF + presaccharification		12.6 ± 2.6	
	CM SSF		16.0 ± 1.9	

Comparison of data obtained from different media and fermentation modes

^1^WE = waste extract from mandis (road-side vegetable markets)

^2^MM = minimal media with 5 g/L glucose

^a^YPG = 20 g peptone, 10 g yeast extract, 60 g glycerol

^b^SCBH = sugarcane bagasse hydrolysate, cSCBH = concentrated SCBH

^c^CPS = carob pulp syrup

^d^SCM = sugarcane molasse

^e^CM = *Camelina* meal

^1,2^Bioreactor, ^a, b, c, d, e^shake flasks. Data from other studies are reported with the original digits and standard deviation

To overcome the need for clarifying the medium after enzymatic hydrolysis, we fed the cells the entire *Camelina* meal hydrolysate, including the water-insoluble solids (WIS) fraction left over after enzymatic hydrolysis. WIS may impair microbial growth and production because of the uneven homogenization of the liquid medium caused by the presence of solid components, as well as due to the toxicity of some of their components [28]. Under conditions termed here as “SSF + presaccharification”, *R. toruloides* was able not only of consuming sugars and producing carotenoids (Fig. 3b), but it also achieved a higher titer of intracellular carotenoids, reaching 13 ± 2.6 mg/L after 96 h, with a yield on consumed sugars of 0.108% (w/w) and on maximum quantity of sugars per biomass of 0.028% (w/w). Given that the amount of carotenoids extracted from *Camelina* meal with and without the addition of enzymes remained constant over time, the carotenoids measured in this and in the following experiments in the presence of WIS were due to microbial metabolism (Additional file 1: Figure S4).

Often proposed as an alternative to SHF, SSF is characterized by a single combined hydrolysis and microbial fermentation step. The two processes have several pros and cons in terms of efficiency, time, presence/release of inhibitory molecules, and downstream final product [42, 43]. SHF and SSF have been proposed and compared for several second-generation biorefineries that used *Arundo donax*, grass, or wheat straw as feedstocks [28, 44, 45]. A potential drawback of incubating enzymes and cells in the same environment is the compromise that needs to be reached allowing optimum operating conditions for both of them. In the present study, because 50 °C was not a viable temperature for *R. toruloides*, 30 °C was selected as the operative temperature. Thus, increased shaking was intended to partially compensate for the reduced activity by augmenting the probability of interactions between the matrix and the enzymes. Remarkably, the release of sugars in these conditions was comparable to that obtained by SHF or SSF + presaccharification (Additional file 1: Figure S5). As shown in Fig. 3c, after the first 6 h of hydrolysis, the amount of sugars in the medium was lower compared to that obtained by SHF, most likely due to the initial growth (and therefore sugar consumption) of *R. toruloides*. After 24 h, sugar consumption became clearly evident and was accompanied by the accumulation of carotenoids. After 96 h, the carotenoid concentration reached 16 ± 1.9 mg/L, with a maximum amount of sugars per biomass of 0.028% (w/w). In the case of SSF, it is not possible to measure the total sugar released during saccharification because of simultaneous fermentation. Importantly, the amount of carotenoids was significantly higher when WIS were left in the medium (SSF and SSF + presaccharification) compared to simple SHF (*t* test *p* < 0.05). While sub-lethal concentrations of insoluble solids might impair microbial growth, they could also trigger the accumulation of metabolites important for the microalgae and yeasts’ own defense systems [41, 46]. For example, β-phenol was shown to trigger carotenoid production in yeast [47].

The titers achieved by SSF indicate the efficacy of concurrent hydrolysis and fermentation, suggesting that a simplified procedure involving a single vessel could be used. Because productivity remained similar over time, the initial sugar released in the presence of cells did not seem to speed up the overall process. Overall, data from SSF and SSF + presaccharification reveal that the often mandatory detoxification step, indicated also for *R. toruloides* [48], is avoidable with this type of residual biomass. Moreover, the final product obtained by both SSF and SSF + presaccharification is a *Camelina* meal enriched with carotenoids, which can be used directly in the animal feed industry.

Therefore, different products, such as pure carotenoids and carotenoids-enriched *Camelina* meal, can be recovered from the tested processes. *Camelina* meal, in particular, would be an innovative product on the market, as carotenoids are commonly added to animal feed for nutritional and organoleptic reasons [3, 12]. In addition, the production of carotenoids from a residual biomass of lower value may increase the economic attractiveness of the proposed process. Based on the logic of cascading [49, 50], the present work paves the way for the use of *Camelina* meal as an alternative feedstock in second-generation biorefineries exploiting microbial cell factories to produce fine chemicals.

## Conclusions

Here, we demonstrate that *Camelina* meal could be employed as residual biomass for the development of novel biorefineries based on microbial cell factories. After enzymatic hydrolysis, this biomass was provided to the oleaginous yeast *R. toruloides* as a sole nutrient and energy source, and carotenoids production was assessed. A comparison of different processes revealed that the highest titer of carotenoids was obtained when *R. toruloides* was exposed to WIS and either SSF (16 ± 1.9 mg/L) or SSF + presaccharification (13 ± 2.6 mg/L). The presence of WIS seemed to play a positive role under these conditions, triggering the accumulation of the desired product and showing how common foes of biorefineries can turn into possible allies. To further investigate the pliancy of this study, we plan to analyze the titer of concurrently accumulated carotenoids (e.g., torulene and torularhodin) and their relative ratio. We also intend to test alternative microbial cell factories to produce other high value-added molecules such as aromas. Moreover, biotransformation will be scaled-up from shake flasks to bioreactors, to generate data useful to calculate the competitiveness of a potential industrial process intended to further valorize *Camelina* meal, following the logic of cascading.

## Materials and methods

### *Camelina* meal composition

Flanat Research Italia S.r.l., Rho, Italy, provided *Camelina* meal derived from plants cultivated and harvested in Lombardy in 2018 and 2019. *C. sativa* seeds were processed to collect the oil, while the leftover meal was delivered to the laboratory and stored at − 20 °C. To measure the water content of *Camelina* meal, 0.9 g and 4.5 g of biomass were dried at 160 °C for 3 h, and then weighted again to calculate the amount of evaporated water. The biomass was incubated at 160 °C for an additional 3 h, but no further changes in weight compared to the value obtained after the initial treatment were observed. Hence, the initial treatment was deemed sufficient. To analyze chemical composition of *Camelina* meal, the biomass was treated following the protocols for the analysis of structural carbohydrates and lignin in biomass from the National Renewable Energy Laboratory (NREL, https://www.nrel.gov/docs/gen/fy13/42618.pdf) with some modifications. In brief, 300 mg of biomass was diluted in 3 mL H_2_SO_4_ 72% (v/v), and then incubated at 30 °C for 1 h, stirring thoroughly every 10 min. The solution was diluted to 4% (v/v) by adding 84 mL of distilled water, mixed by inversion, and autoclaved at 121 °C for 1 h. The hydrolysis solution was vacuum-filtered through a previously weighted filtering crucible, and the insoluble components were measured gravimetrically on the filter paper. The filtered liquid was neutralized with NaOH until pH 5–6 was attained and then, the samples were analyzed by HPLC (as described below) after filtration with a 0.22-µm filter (Euroclone, Pero, Milan, Italy). Three independent experiments were performed.

### Pretreatment and enzymatic hydrolysis of *Camelina* meal

Enzymatic hydrolysis of *Camelina* meal was performed using the enzyme mixture NS22119, kindly provided by Novozymes (Novozymes A/S, Copenhagen, Denmark). As described by the producer, NS22119 contains a wide range of carbohydrases, including arabinase, β-glucanase, cellulase, hemicellulase, pectinase, and xylanase from *Aspergillus aculeatus*. Without drying the biomass, different quantities of *Camelina* meal were weighted to a concentration of 3%, 6%, 9%, 12%, and 15% (w/v) into glass bottles, steeped in water with a final volume of 30 mL, and then autoclaved at 121 °C for 1 h to both sterilize and pre-treat the biomass. Afterward, enzymes were added directly to the bottles and incubated at pH 5.5 and at 50 °C in a water bath under mild agitation (105 rpm). A 1-mL aliquot was collected every 2, 4, 6, and 24 h from the start, and sugar content was analyzed by HPLC (see below). The following enzyme concentrations were tested: 11.9% w/w_*Camelina* meal_, 23.8% w/w_*Camelina* meal_, and 11.9% w/w_*Camelina* meal_ at 0 h plus at 6 h. Three independent experiments were performed. Low enzyme doses were intended to mimic commercially feasible hydrolysis, whereas high doses provided an indication of maximum enzymatically accessible sugar content. When implementing the suggested process at commercial scale, additional testing is recommended to refine the dose–response curve and determine the effect of time, solids loading, pretreatment protocol, cellulose conversion, and enzyme dosage.

### Microbial strain and media

*Rhodosporidium toruloides* (DSM 4444) was purchased from DSMZ (German Collection of Microorganisms and Cell Cultures, GmbH) and stored in cryotubes at − 80 °C in 20% glycerol (v/v). The composition of the medium for the pre-inoculum was as follows (per liter): 1 g yeast extract, 1.31 g (NH_4_)_2_SO_4_, 0.95 g Na_2_HPO_4_, 2.7 g KH_2_PO_4_, and 0.2 g Mg_2_SO_4_·7H_2_O. The medium was supplemented with 15 g/L of glycerol as main carbon source and a 100× trace mineral stock solution consisting of (per liter) 4 g CaCl_2_‧2H_2_O, 0.55 g FeSO_4_·7H_2_O, 0.52 g citric acid, 0.10 g ZnSO_4_·7H_2_O, 0.076 g MnSO_4_·H_2_O, and 100 μL 18 M H_2_SO_4_. Yeast extract was purchased from Biolife Italia S.r.l., Milan, Italy. All other reagents were purchased from Sigma-Aldrich Co., St Louis, MO, USA. After plating on rich medium, a pre-inoculum was run in rich medium until stationary phase. Then, cells were inoculated at 0.2 OD in shake flasks at 30 °C and 160 rpm for both SHF and SSF processes (see below).

### SHF and SSF

During both SHF and SSF processes, *R. toruloides* was grown in shake flasks at pH 5.5, supplemented with *Camelina* meal hydrolysate, with or without WIS. After 6 h of enzymatic hydrolysis at 50 °C, the hydrolysate was centrifuged at 4000 rpm for 10 min to separate the water-soluble components from WIS. Then, for SHF, the liquid fraction was collected and transferred into a shake flask for microbial growth at 30 °C. Alternatively, for the SSF + saccharification process, *Camelina* hydrolysate was provided directly to *R. toruloides* as growth medium, regardless of the presence of WIS. For the SSF process, *Camelina* meal was directly steeped and autoclaved in a shake flask, then supplemented with the enzymatic cocktail at 11.9% w/w_*Camelina* meal_ and 0.2 OD of cells, and incubated at 30 °C and 160 rpm. Three independent experiments for each setting were performed.

### Carotenoids extraction

Carotenoids were analyzed by acetone extraction from *R. toruloides* cells with a protocol adapted from [51]. In brief, 1 mL of culture broth was collected and harvested by centrifugation at 7000 rpm for 7 min at 4 °C, and the pellet was then resuspended in 1 mL acetone and broken using glass beads by thorough agitation with a FastPrep-24™ (MP Biomedicals, LLC, Santa Ana, CA, USA). Carotenoids were extracted in the acetone phase, the suspension was centrifuged, and the supernatant collected. The extraction was repeated with fresh acetone until the biomass was colorless. Carotenoid content was measured spectrophotometrically (see below).

### Analytical methods

HPLC analyses were performed to quantify the amount of glucose, sucrose, arabinose, fructose, and acetic acid. In brief, 1-mL samples from each of the three different streams of production (enzymatically hydrolyzed *Camelina* meal, SHF or SSF) were collected and centrifuged twice (7000 rpm, 7 min, and 4 °C), and then analyzed by HPLC using a Rezex ROA-Organic Acid column (Phenomenex, Torrance, CA, USA). The eluent was 0.01 M H_2_SO_4_ pumped at 0.5 mL/min and column temperature was 35 °C. Separated components were detected by a refractive index detector and peaks were identified by comparing with known standards (Sigma-Aldrich). Optical density (OD) of *R. toruloides* was measured spectrophotometrically at 600 nm. The pH was measured with indicator strips at the beginning and at the end of enzymatic hydrolysis to assess suitability of the initial conditions and to foresee possible toxic effects of the final medium.

The titer of carotenoids extracted in acetone from *R. toruloides* was determined spectrophotometrically (UV-1800; Shimadzu, Kyoto, Japan) based on the maximum absorption peak for β-carotene (455 nm). A calibration curve with standard concentration of β-carotene was obtained.

### Calculations and statistical analyses

Sugar recovery (here *S*_r_) was calculated as percentage of sugar yield obtained by enzymatic hydrolysis (here *Y*_EH_) compared with the yield obtained from total acid hydrolysis of biomass (here *Y*_AH_) (Eq. 1). Carotenoids yield on consumed sugars (here *Y*_c/s_) and carotenoids yield on maximum quantity of sugars per biomass (here *Y*_c/b_) measured with acid hydrolysis were calculated by Eqs. 2 and 3, respectively.

1 $$S_{\text{r}} = {\raise0.7ex\hbox{${Y_{\text{EH}} }$} \!\mathord{\left/ {\vphantom {{Y_{\text{EH}} } {Y_{\text{AH}} }}}\right.\kern-0pt} \!\lower0.7ex\hbox{${Y_{\text{AH}} }$}} \times 100$$

2 $$Y_{{{\text{c}}/{\text{s}}}} = {\raise0.7ex\hbox{${C_{\text{p}} }$} \!\mathord{\left/ {\vphantom {{C_{\text{p}} } {\Delta {\text{sug}}}}}\right.\kern-0pt} \!\lower0.7ex\hbox{${\Delta {\text{sug}}}$}} \times 100$$

3 $$Y_{{{\text{c}}/{\text{b}}}} = {\raise0.7ex\hbox{${C_{\text{p}} }$} \!\mathord{\left/ {\vphantom {{C_{\text{p}} } {S_{\text{b}} }}}\right.\kern-0pt} \!\lower0.7ex\hbox{${S_{\text{b}} }$}} \times 100$$

where Δsug corresponds to consumed sugars, *S*_b_ to maximum quantity of sugars in the biomass, and *C*_p_ to carotenoids produced.

For statistical analysis, heteroscedastic two-tailed *t* test was applied.

## Supplementary information

**Additional file 1: Figure S1.** Effect of enzymatic hydrolysis conditions on different concentrations of *Camelina* meal without addition of the NS22119 cocktail. **Figure S2.** Acetic acid released during enzymatic hydrolysis. The concentration of acetic acid released from 15% *Camelina* meal by treatment with the NS22119 cocktail (11.9% w/w_*Camelina* meal_) was evaluated over time. **Figure S3.***R. toruloides* production of carotenoids from 15% (w/v) *Camelina* meal hydrolysate. OD (dotted line), sugars consumption (dashed line), and β-carotene production (white bars) by *R. toruloides* during the SHF process are shown. **Figure S4.** Carotenoids’ extraction from *Camelina* meal hydrolysate. **Figure S5.** Effect of enzymatic hydrolysis on 15% *Camelina* meal by the NS22119 cocktail (11.9% w/w_*Camelina* meal_) at 30 °C.

## Data Availability

Not applicable.

## Abbreviations

HPLC: High-performance liquid chromatography
OD: Optical density
SHF: Separate hydrolysis and fermentation
SSF: Simultaneous saccharification and fermentation
WIS: Water-insoluble solids

## References

[1] Azapagic A (2014). Sustainability considerations for integrated biorefineries. Trends Biotechnol.

[2] Jönsson LJ, Martín C (2016). Pretreatment of lignocellulose: formation of inhibitory by-products and strategies for minimizing their effects. Bioresour Technol.

[3] Food and Agriculture Organization of the United Nations (FAO). Utilization of lipid co-products of the biofuel industry in livestock feed in Biofuel co-products as livestock feed; 2012.

[4] Zubr J (2010). Carbohydrates, vitamins and minerals of *Camelina sativa* seed. Nutr Food Sci.

[5] Cherian G (2012). *Camelina sativa* in poultry diets : opportunities and challenges Biofuel co-products as livestock feed: opportunities and challenges.

[6] Murphy EJ (2016). Camelina (*Camelina sativa*). Industrial oil crops.

[7] Dharavath RN, Singh S, Chaturvedi S, Luqman S (2016). *Camelina sativa* (L.) Crantz A mercantile crop with speckled pharmacological activities. Ann Phytomed.

[8] Sizmaz O, Gunturkun OB, Zentek J (2016). A point on nutritive value of *Camelina* meal for broilers: a review. Int J Vet Sci.

[9] Li X, Mupondwa E. Production and value-chain integration of *Camelina sativa* as a dedicated bioenergy feedstock in the Canadian prairies. In: 24th European biomass conference & exhibition, Amsterdam Netherlands, 2016.

[10] Mohammad BT, Al-Shannag M, Alnaief M, Singh L, Singsaas E, Alkasrawi M (2018). Production of multiple biofuels from whole *Camelina* material: a renewable energy crop. BioResources.

[11] Wang C, Zhao S, Shao X, Park JB, Jeong SH, Park HJ (2019). Challenges and tackles in metabolic engineering for microbial production of carotenoids. Microb Cell Fact.

[12] Saini RK, Keum Y-S (2019). Microbial platforms to produce commercially vital carotenoids at industrial scale: an updated review of critical issues. J Ind Microbiol Biotechnol.

[13] Yuan S-F, Alper HS (2019). Metabolic engineering of microbial cell factories for production of nutraceuticals. Microb Cell Fact.

[14] Nagarajan J, Ramanan RN, Raghunandan ME, Galanakis CM, Krishnamurthy NP (2017). Carotenoids. Nutraceutical and functional food components.

[15] Guedes AC, Amaro HM, Malcata FX (2011). Microalgae as sources of carotenoids. Mar Drugs.

[16] Lee JJL, Chen L, Shi J, Trzcinski A, Chen WN (2014). Metabolomic profiling of *Rhodosporidium toruloides* grown on glycerol for carotenoid production during different growth phases. J Agric Food Chem.

[17] Frengova GI, Beshkova DM (2009). Carotenoids from *Rhodotorula* and *Phaffia*: yeasts of biotechnological importance. J Ind Microbiol Biotechnol.

[18] Kot AM, Błazejak S, Gientka I, Kieliszek M, Bryś J (2018). Torulene and torularhodin: “New” fungal carotenoids for industry?.

[19] Park YK, Nicaud JM, Ledesma-Amaro R (2018). The engineering potential of *Rhodosporidium toruloides* as a workhorse for biotechnological applications. Trends Biotechnol.

[20] Sitepu I, Selby T, Lin T, Zhu S, Boundy-Mills K (2014). Carbon source utilization and inhibitor tolerance of 45 oleaginous yeast species. J Ind Microbiol Biotechnol.

[21] Bonturi N, Crucello A, Viana AJC, Miranda EA (2017). Microbial oil production in sugarcane bagasse hemicellulosic hydrolysate without nutrient supplementation by a *Rhodosporidium toruloides* adapted strain. Process Biochem.

[22] Freitas C, Parreira TM, Roseiro J, Reis A, Da Silva TL (2014). Selecting low-cost carbon sources for carotenoid and lipid production by the pink yeast *Rhodosporidium toruloides* NCYC 921 using flow cytometry. Bioresour Technol.

[23] Singh G, Sinha S, Bandyopadhyay KK, Lawrence M, Paul D (2018). Triauxic growth of an oleaginous red yeast *Rhodosporidium toruloides* on waste “extract” for enhanced and concomitant lipid and β-carotene production. Microb Cell Fact.

[24] Dai X, Shen H, Li Q, Rasool K, Wang Q, Yu X (2019). Microbial lipid production from corn stover by the oleaginous yeast *Rhodosporidium toruloides* using the PreSSLP process. Energies.

[25] Wahlström RM, Suurnäkki A (2015). Enzymatic hydrolysis of lignocellulosic polysaccharides in the presence of ionic liquids. Green Chem.

[26] Khare SK, Pandey A, Larroche C (2015). Current perspectives in enzymatic saccharification of lignocellulosic biomass. Biochem Eng J.

[27] Arevalo-Gallegos A, Ahmad Z, Asgher M, Parra-Saldivar R, Iqbal HMN (2017). Lignocellulose: a sustainable material to produce value-added products with a zero waste approach—a review. Int J Biol Macromol.

[28] Ask M, Olofsson K, Di Felice T, Ruohonen L, Penttilä M, Lidén G (2012). Challenges in enzymatic hydrolysis and fermentation of pretreated *Arundo donax* revealed by a comparison between SHF and SSF. Process Biochem.

[29] Kim JK, Yang J, Park SY, Yu JH, Kim KH (2019). Cellulase recycling in high-solids enzymatic hydrolysis of pretreated empty fruit bunches. Biotechnol Biofuels.

[30] Dias C, Sousa S, Caldeira J, Reis A, da Silva TL (2015). New dual-stage pH control fed-batch cultivation strategy for the improvement of lipids and carotenoids production by the red yeast *Rhodosporidium toruloides* NCYC 921. Bioresour Technol.

[31] Merino ST, Cherry J (2007). Progress and challenges in enzyme development for biomass utilization. Adv Biochem Eng Biotechnol.

[32] Lenihan P, Orozco A, O’Neill E, Ahmad MNM, Rooney DW, Walker GM (2010). Dilute acid hydrolysis of lignocellulosic biomass. Chem Eng J.

[33] Sousa MJ, Ludovico P, Rodrigues F, Leo C, Crte-Real M (2012). Stress and cell death in yeast induced by acetic acid. Cell metabolism—cell homeostasis and stress response.

[34] Martani F, Marano F, Bertacchi S, Porro D, Branduardi P (2015). The *Saccharomyces cerevisiae* poly(A) binding protein Pab1 as a target for eliciting stress tolerant phenotypes. Sci Rep.

[35] Guldfeldt LU, Arneborg N (1998). Measurement of the effects of acetic acid and extracellular pH on intracellular pH of nonfermenting, individual *Saccharomyces cerevisiae* cells by fluorescence microscopy. Appl Environ Microbiol.

[36] Pampulha ME, Loureiro-Dias MC (2000). Energetics of the effect of acetic acid on growth of *Saccharomyces cerevisiae*. FEMS Microbiol Lett.

[37] Zhao X, Peng F, Du W, Liu C, Liu D (2012). Effects of some inhibitors on the growth and lipid accumulation of oleaginous yeast *Rhodosporidium toruloides* and preparation of biodiesel by enzymatic transesterification of the lipid. Bioprocess Biosyst Eng.

[38] Huang XF, Liu JN, Lu LJ, Peng KM, Yang GX, Liu J (2016). Culture strategies for lipid production using acetic acid as sole carbon source by *Rhodosporidium toruloides*. Bioresour Technol.

[39] Hu C, Zhao X, Zhao J, Wu S, Zhao ZK (2009). Effects of biomass hydrolysis by-products on oleaginous yeast *Rhodosporidium toruloides*. Bioresour Technol.

[40] Singh G, Jawed A, Paul D, Bandyopadhyay KK, Kumari A, Haque S (2016). Concomitant production of lipids and carotenoids in *Rhodosporidium toruloides* under osmotic stress using response surface methodology. Front Microbiol.

[41] Mata-Gómez LC, Montañez JC, Méndez-Zavala A, Aguilar CN (2014). Biotechnological production of carotenoids by yeasts: an overview. Microb Cell Fact.

[42] Aditiya HB, Mahlia TMI, Chong WT, Nur H, Sebayang AH (2016). Second generation bioethanol production: a critical review. Renew Sustain Energy Rev.

[43] Srivastava N, Rawat R, Singh Oberoi H, Ramteke PW (2015). A review on fuel ethanol production from lignocellulosic biomass. Int J Green Energy.

[44] Burman NW, Sheridan CM, Harding KG (2019). Lignocellulosic bioethanol production from grasses pre-treated with acid mine drainage: modeling and comparison of SHF and SSF. Bioresour Technol Rep.

[45] Tomás-Pejó E, Oliva JM, Ballesteros M, Olsson L (2008). Comparison of SHF and SSF processes from steam-exploded wheat straw for ethanol production by xylose-fermenting and robust glucose-fermenting *Saccharomyces cerevisiae* strains. Biotechnol Bioeng.

[46] Sun X-M, Ren L-J, Zhao Q-Y, Ji X-J, Huang H (2018). Microalgae for the production of lipid and carotenoids: a review with focus on stress regulation and adaptation. Biotechnol Biofuels.

[47] Kim BK, Park PK, Chae HJ, Kim EY (2004). Effect of phenol on β-carotene content in total carotenoids production in cultivation of *Rhodotorula glutinis*. Korean J Chem Eng.

[48] Matsakas L, Novak K, Enman J, Christakopoulos P, Rova U (2017). Acetate-detoxification of wood hydrolysates with alkali tolerant *Bacillus* sp. as a strategy to enhance the lipid production from *Rhodosporidium toruloides*. Bioresour Technol.

[49] IEA Bioenergy Task42. Sustainable and synergetic processing of biomass into marketable food & feed ingredients, chemicals, materials and energy (fuels, power, heat). IEA Bioenergy. 2014;66.

[50] IEA Bioenergy Task40. Cascading of woody biomass: definitions, policies and effects on international trade. IEA Bioenergy. 2016;71.

[51] Saenge C, Cheirsilp B, Suksaroge TT, Bourtoom T (2011). Potential use of oleaginous red yeast *Rhodotorula glutinis* for the bioconversion of crude glycerol from biodiesel plant to lipids and carotenoids. Process Biochem.

