# Supplementary material for: Camelina sativa meal hydrolysate as sustainable biomass for the production of carotenoids by Rhodosporidium toruloides
Source: Biotechnol Biofuels. 2020 Mar 12;13:47. doi: 10.1186/s13068-020-01682-3 (PMC7066749; doi:10.1186/s13068-020-01682-3)
Supplement: Supplementary file 1 — Additional file 1: Figure S1. Effect of enzymatic hydrolysis conditions on different concentrations of Camelina meal without addition of the NS22119 cocktail. Figure S2. Acetic acid released during enzymatic hydrolysis. The concentration of acetic acid released from 15% Camelina meal by treatment with the NS22119 cocktail (11.9% w/wCamelina meal) was evaluated over time. Figure S3.R. toruloides production of carotenoids from 15% (w/v) Camelina meal hydrolysate. OD (dotted line), sugars consumption (dashed line), and β-carotene production (white bars) by R. toruloides during the SHF process are shown. Figure S4. Carotenoids’ extraction from Camelina meal hydrolysate. Figure S5. Effect of enzymatic hydrolysis on 15% Camelina meal by the NS22119 cocktail (11.9% w/wCamelina meal) at 30 °C. [file 13068_2020_1682_MOESM1_ESM.pptx]

## Slide 1
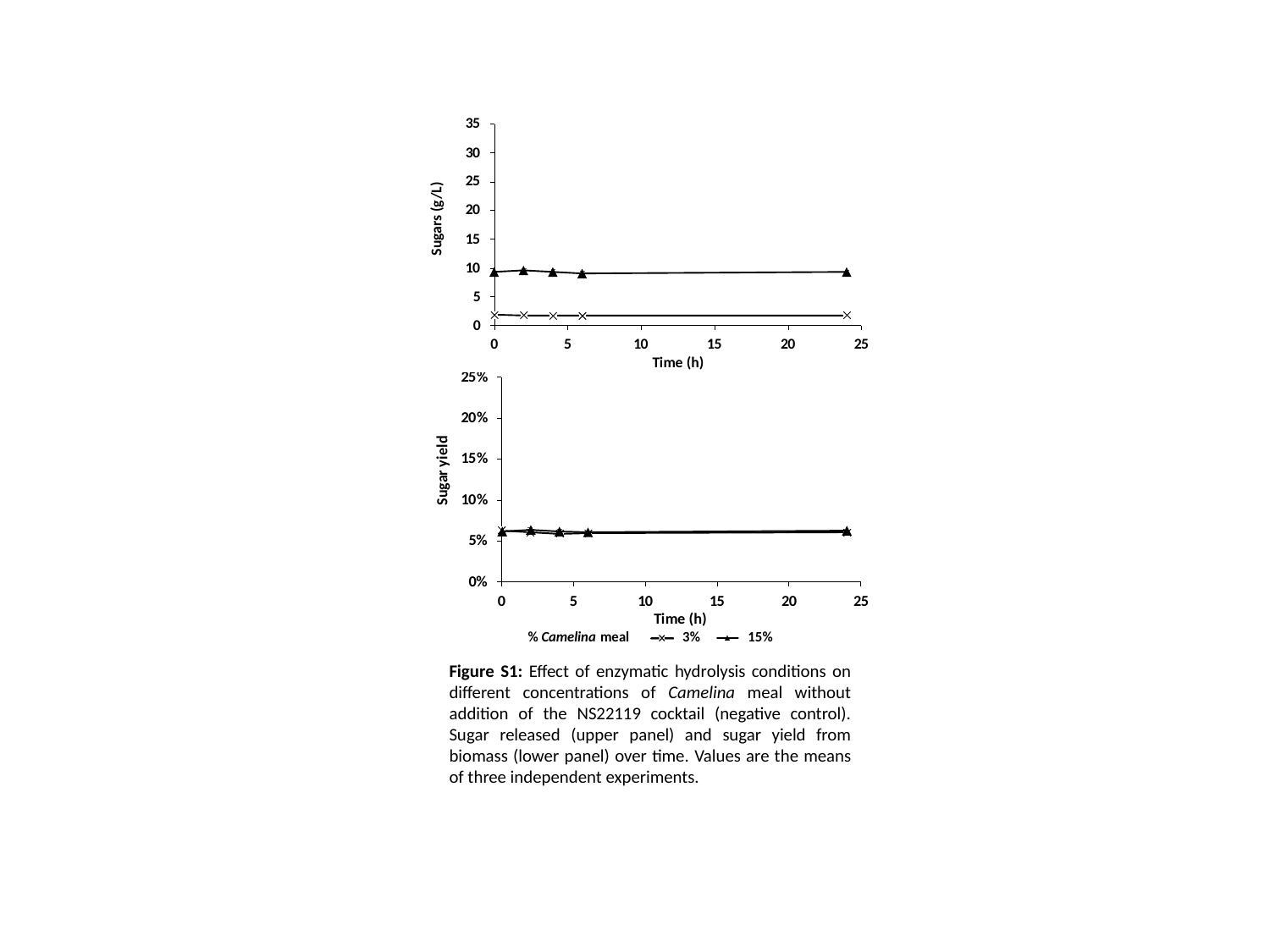

Figure S1: Effect of enzymatic hydrolysis conditions on different concentrations of Camelina meal without addition of the NS22119 cocktail (negative control). Sugar released (upper panel) and sugar yield from biomass (lower panel) over time. Values are the means of three independent experiments.

## Slide 2
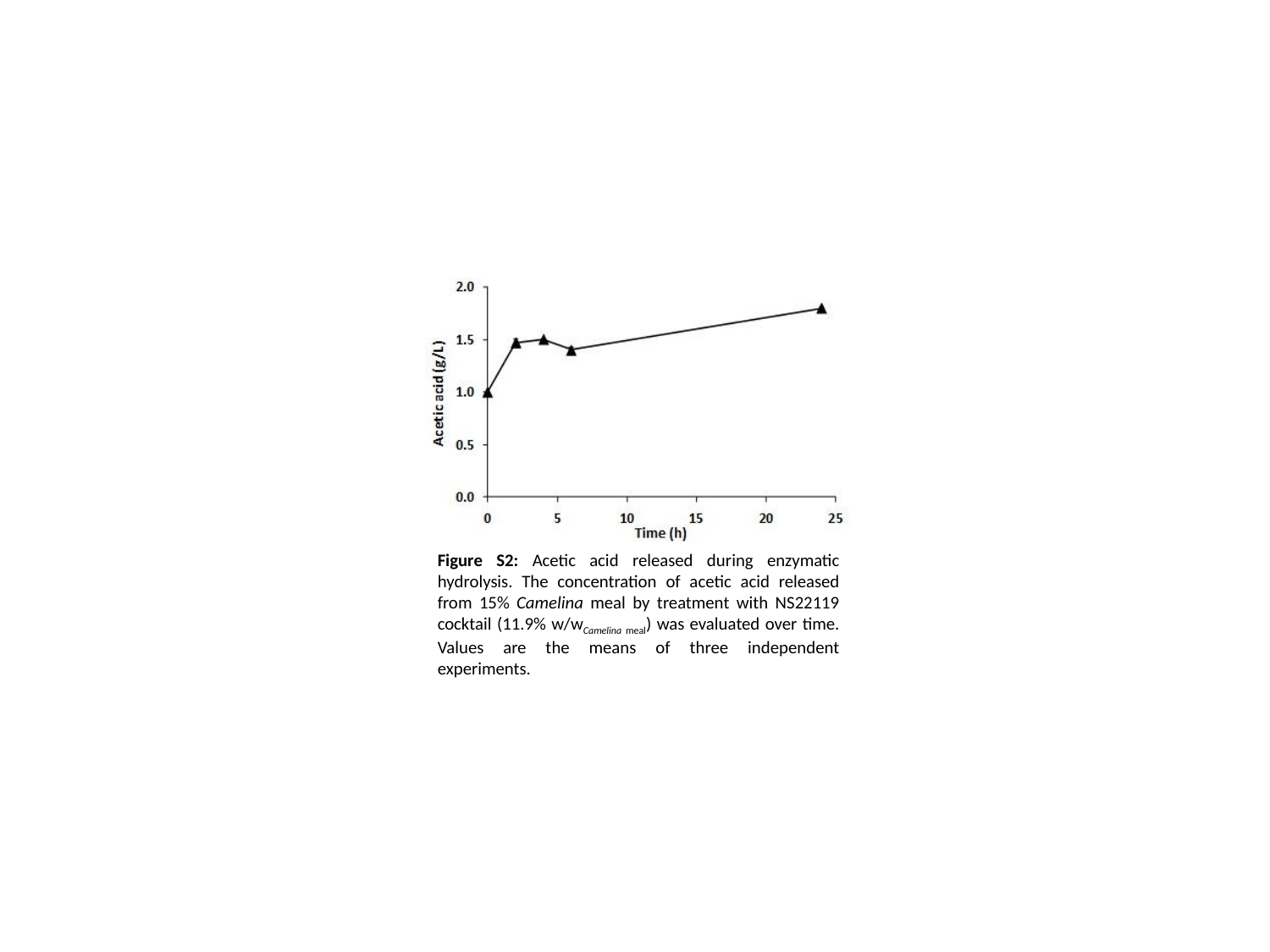

Figure S2: Acetic acid released during enzymatic hydrolysis. The concentration of acetic acid released from 15% Camelina meal by treatment with NS22119 cocktail (11.9% w/wCamelina meal) was evaluated over time. Values are the means of three independent experiments.

## Slide 3
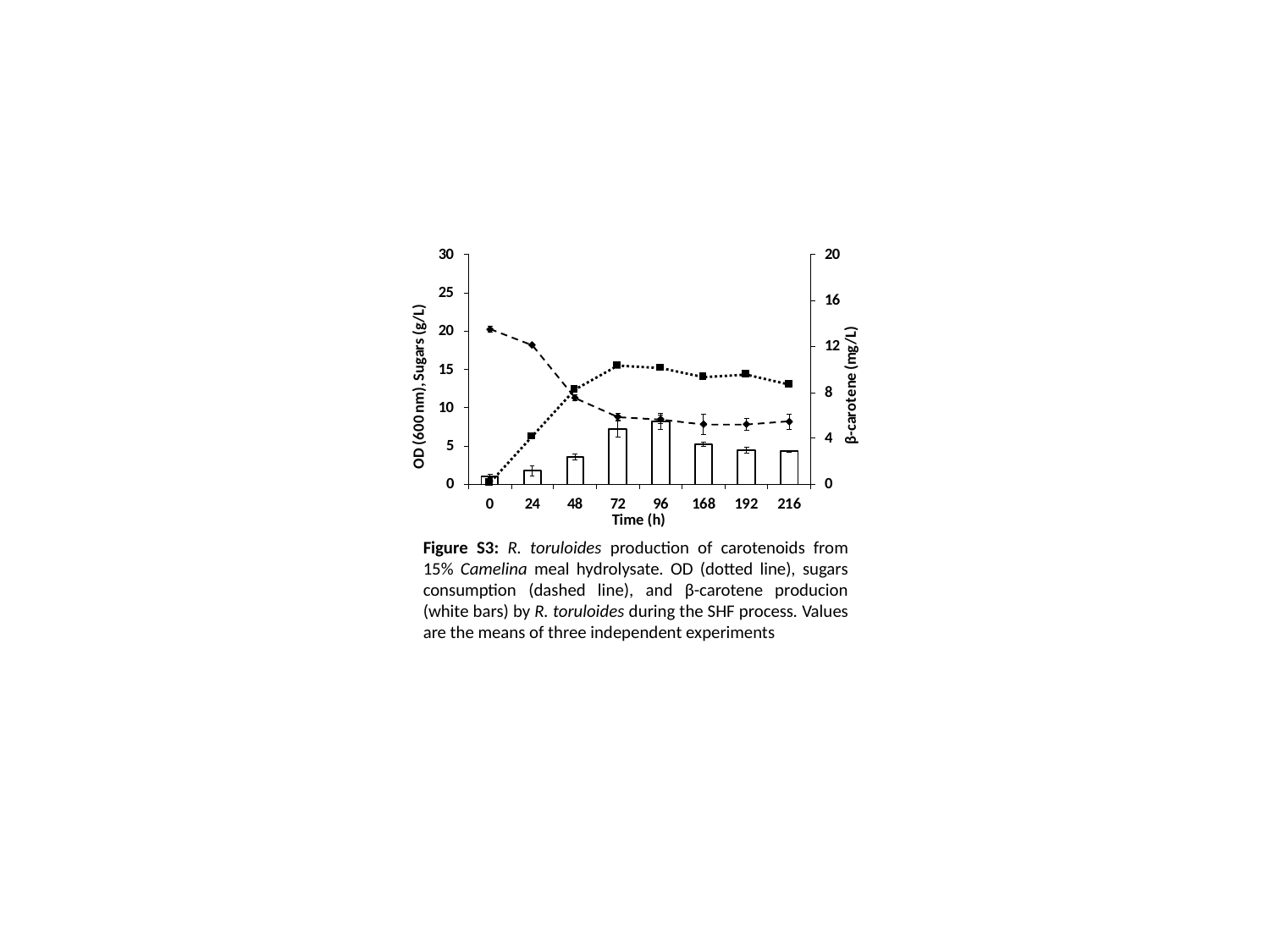

Figure S3: R. toruloides production of carotenoids from 15% Camelina meal hydrolysate. OD (dotted line), sugars consumption (dashed line), and β-carotene producion (white bars) by R. toruloides during the SHF process. Values are the means of three independent experiments

## Slide 4
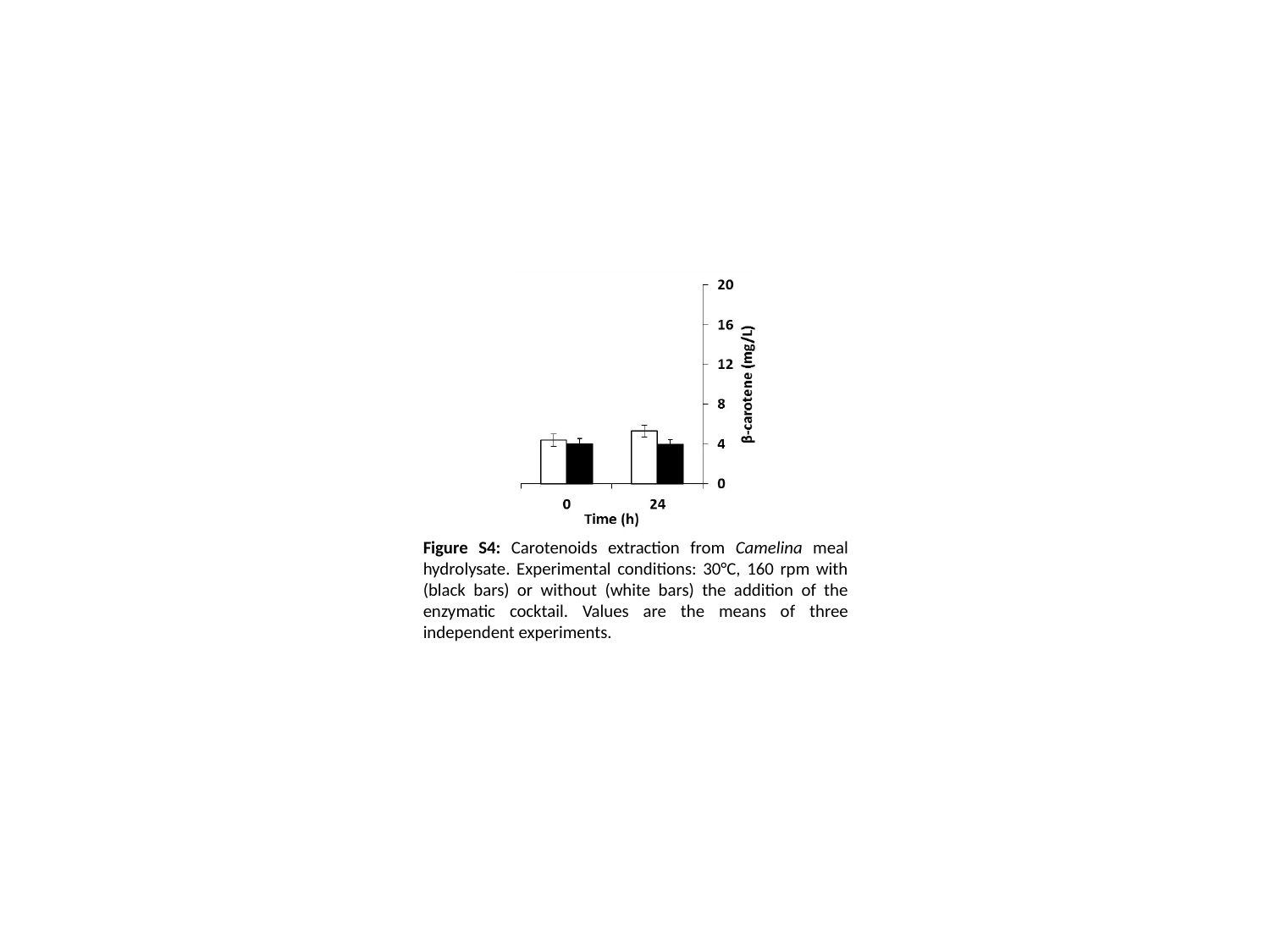

Figure S4: Carotenoids extraction from Camelina meal hydrolysate. Experimental conditions: 30°C, 160 rpm with (black bars) or without (white bars) the addition of the enzymatic cocktail. Values are the means of three independent experiments.

## Slide 5
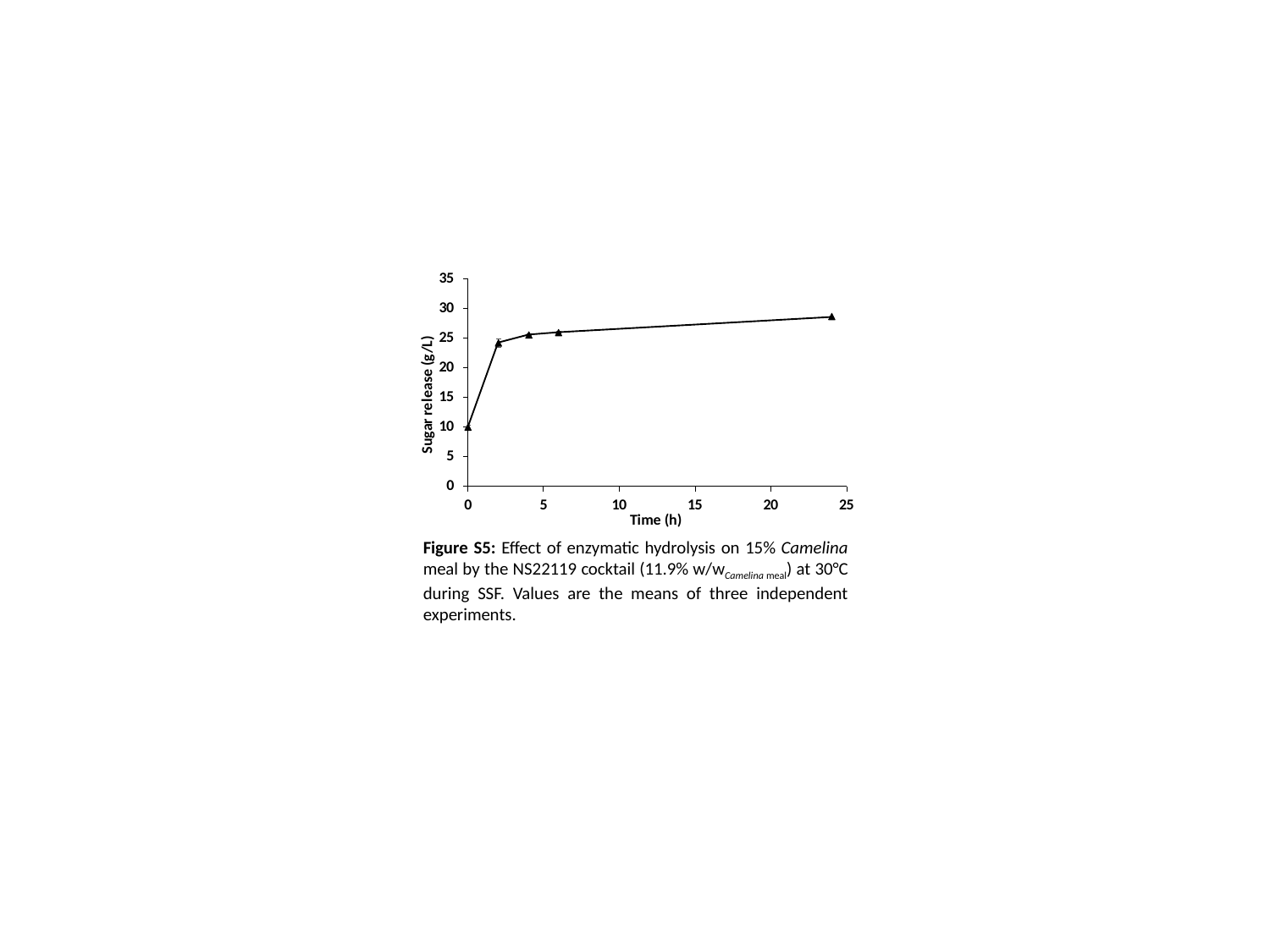

Figure S5: Effect of enzymatic hydrolysis on 15% Camelina meal by the NS22119 cocktail (11.9% w/wCamelina meal) at 30°C during SSF. Values are the means of three independent experiments.
